# Supplementary material for: Smart Clothing as a Noninvasive Method to Measure the Physiological Cardiac Parameters
Source: Healthcare (Basel). 2021 Oct 2;9(10):1318. doi: 10.3390/healthcare9101318 (PMC8544566; doi:10.3390/healthcare9101318)
Supplement: Supplementary file 1 [file healthcare-09-01318-s001.zip › healthcare-1339223-supplementary.pdf]

healthcare

MDPI

Supplementary information

Table S1 ANOVA for subject's independent variables vesus heartbeat-difference

	Df	Sum Sq	Mean Sq	F value	Pr (>F)
Gender	1	0.729	0.729	0.549	0.492
Age	2	3.337	1.668	1.258	0.361
Gender:Age	1	2.51	2.51	1.892	0.227
Residuals	5	6.633	1.327		

※ Significant P < 0.05. No significant difference detected in this test

Table S2 Bayesian ANOVA for subject's independent variables

Formula	Effect	Error
[1] Gender	0.568588	±0%
[2] Age	0.718174	±0.01%
[3] Gender + Age	0.386449	±1.03%
[4] Gender + Age + Gender:Age	0.412438	±2.11%

※ Significant effect > 1. No significant difference detected in this test

Table S3 The summary of the Student t-test for HB data

		Paired differences						
				95% confidence interval of the difference				
	Mean	Standard deviation	Standard error of the mean	Lower limit	Upper limit	t	df	Significance (two-tailed)
Pair 1 clothbpm - jelbpm	0.01000	1.21147	0.38310	-0.85664	0.87664	0.026	9	0.980

Table S4 R code for processing Bayesian t-test for HB data

```
> library(BayesFactor)
> HB <- read.table("HeartBeat.txt", header=TRUE)
> HB_ttestBF <- ttestBF(x=HB$Suite, y=HB$Gel, paired=TRUE, posterior=TRUE, iterations=1000)
```

Table S5 Output of testBFfor HB data

```
Iterations = 1:1000
Thinning interval = 1
Number of chains = 1
Sample size per chain = 1000

1. Empirical mean and standard deviation for each variable,
   plus standard error of the mean:

            Mean       SD  Naive SE Time-series SE
mu     -0.001977   0.3569  0.011285       0.010733
sig2    1.694825   1.0303  0.032580       0.036417
delta   0.001678   0.2772  0.008765       0.008765
g      14.591797 383.0622 12.113491      12.113491

2. Quantiles for each variable:
```

	2.5%	25%	50%	75%	97.5%
mu	−0.70214	−0.2178	0.009215	0.2168	0.6768
sig2	0.67303	1.0774	1.435597	2.0004	4.2836
delta	−0.53435	−0.1800	0.006335	0.1839	0.5533
g	0.07194	0.1972	0.408382	0.9824	13.2841
